# Growth and Self-Assembly of CsPbBr_3_ Nanocrystals in the TOPO/PbBr_2_ Synthesis as Seen with X-ray Scattering

**DOI:** 10.1021/acs.nanolett.2c04532

**Published:** 2023-01-06

**Authors:** Federico Montanarella, Quinten A. Akkerman, Dennis Bonatz, Maaike M. van der Sluijs, Johanna C. van der Bok, P. Tim Prins, Marcel Aebli, Alf Mews, Daniel Vanmaekelbergh, Maksym V. Kovalenko

**Affiliations:** † Laboratory of Inorganic Chemistry, Department of Chemistry and Applied Biosciences, ETH Zürich, Vladimir Prelog Weg 1, CH-8093Zürich, Switzerland; ‡ Laboratory for Thin Films and Photovoltaics, Empa − Swiss Federal Laboratories for Materials Science and Technology, Überlandstrasse 129, CH-8600Dübendorf, Switzerland; § Debye Institute for Nanomaterials Science, Utrecht University, 3584 CCUtrecht, The Netherlands; ∥ Institute of Physical Chemistry, University of Hamburg, 20146Hamburg, Germany

**Keywords:** lead halides, perovskites, nanocrystals, growth, *in situ*, quantum dots, self-assembly, X-ray scattering

## Abstract

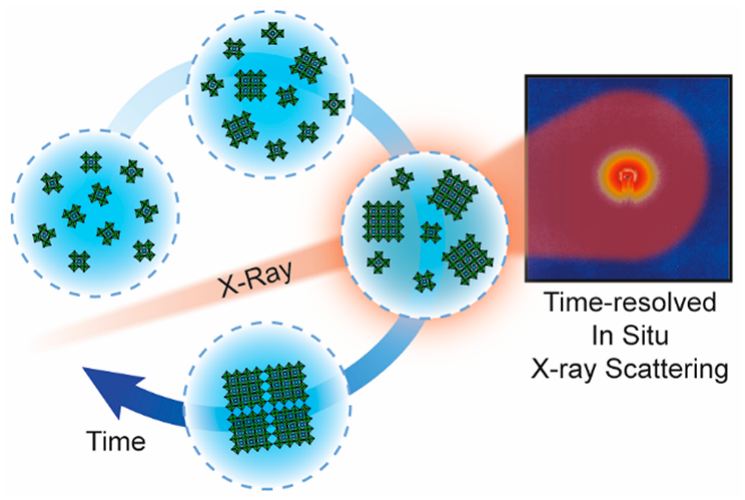

Despite broad interest in colloidal lead halide perovskite nanocrystals (LHP NCs), their intrinsic fast growth has prevented controlled synthesis of small, monodisperse crystals and insights into the reaction mechanism. Recently, a much slower synthesis of LHP NCs with extreme size control has been reported, based on diluted TOPO/PbBr_2_ precursors and a diisooctylphosphinate capping ligand. We report new insights into the nucleation, growth, and self-assembly in this reaction, obtained by *in situ* synchrotron-based small-angle X-ray scattering and optical absorption spectroscopy. We show that dispersed 3 nm Cs[PbBr_3_] agglomerates are the key intermediate species: first, they slowly nucleate into crystals, and then they release Cs[PbBr_3_] monomers for further growth of the crystals. We show the merits of a low Cs[PbBr_3_] monomer concentration for the reaction based on oleate ligands. We also examine the spontaneous superlattice formation mechanism occurring when the growing nanocrystals in the solvent reach a critical size of 11.6 nm.

Ligand-capped colloidal nanocrystals (NCs) of lead halide perovskite (LHP), especially cesium lead halide (CsPbX_3_; X = Cl, Br, I), are the latest generation of visible-light-emissive semiconductor NCs. So far, they have been produced via facile and fast ionic coprecipitation. They exhibit near-unity photoluminescence efficiency, even without surface shelling for electronic passivation, and high defect tolerance.^1−6^ These NCs have attracted attention as single-photon emitters^7−10^ and also feature aggregated emissive states.^11−15^ LHP NCs are actively pursued for diverse applications, foremost in light-emitting diodes^16−18^ and downconversion displays,^19−21^ as well as scintillators,^22−24^ photodetectors,^22,25−29^ printed security tags,^30,31^ etc. Despite their accelerated practical deployment, the literature on the synthesis of LHP NCs continues to expand; the variants of diverse synthesis protocols keep increasing, differing in, for instance, the precursors, ligand chemistry, solvents, NC surface treatments, and other postsynthetic processing steps.^32−44^ By and large, the motivation behind these intense synthesis efforts is rooted in the challenges that arise from the inherently soft and labile nature of these ionic semiconductors.

From early on, the inherently fast kinetics of the ionic coprecipitation reactions was the major hurdle for size control, especially in the small-size regime of strong quantum confinement. Specifically, the common reactions, such as the standard oleic acid–oleylamine hot-injection synthesis (OA/OlAm),^1^ are nearly instantaneous, since relevant cationic (Cs^+^) and anionic (PbBr_3_^–^) species are present from the beginning. Typical protocols that rely on mixing liquids in practically relevant quantities (milliliter to liter) are therefore characterized by very fast nucleation and growth rates. In these cases, state-of-the-art *in situ* optical absorption spectroscopy and X-ray scattering methods, capable of handling millisecond time scales,^45,46^ still yield unsatisfactory outcomes. The challenge of resolving the early formation stage of perovskite NCs was not even fully mitigated when the same OA/OlAm synthesis was conducted in nanoliter-sized droplets using microfluidic platforms that assured mixing-to-homogenization times of under 300 ms and a mixing-to-optical-spectrum-acquisition time under 100 ms.^47,48^ Self-evidently, synthesis methods relying on very fast reactions lack the option of isolating a desired NC size fraction by controlling the growth time, a common convenient practice for other semiconductor NCs.^49,50^

We recently introduced a room-temperature synthesis of CsPbBr_3_ NCs with growth times of up to 30 min.^51^ Such a drastic reduction in the reaction rates primarily arises from the intricate solution equilibria involving the precursors (PbBr_2_ coordinated by trioctylphosphine oxide (TOPO) and Cs-DOPA (DOPA = diisooctylphosphinate)), the monomer intermediates, i.e., Cs[PbBr_3_] species coordinated by TOPO (see below), and the resulting CsPbBr_3_ NCs and other formed Pb species (Pb(DOPA)_2_). Importantly, in the absence of amines and acids in the Pb precursor solution, the addition of Cs-DOPA specifically causes the formation of the equivalent quantity of bromoplumbate (PbBr_3_^–^) and Cs[PbBr_3_] species. The new method resulted in an unprecedented resolution of excitonic features (with up to three higher excitonic transitions) across a broad range of NC sizes (3–14 nm), stemming from both narrow size dispersion and nearly spherical (i.e. rhombicuboctahedral) shapes.^51,52^*In situ* absorption spectroscopy was highly instrumental for tracing the evolution of NCs and depletion of precursors, enabling us to study equilibria essential for the NC formation kinetics. To fulfill the technical requirements of the *in situ* absorption spectroscopy (optical densities matching the dynamic range of detectors), the TOPO/PbBr_2_ synthesis was optimized to yield CsPbBr_3_ NC concentrations substantially lower than those typically used for perovskite and other semiconductor NCs (molarity of deficient species: ca. (0.1–0.5) × 10^–3^ M versus ca. (7–12) × 10^–3^ M,^1,53^ and ca. (5–10) × 10^–2^ M.^54,55^). We also note that absorption spectroscopy alone might not be sufficient to obtain direct information on the size and morphology of dispersed particles, on the nucleating and growing NCs or on their uniformity and temporal evolution during the synthesis. These tasks are uniquely matched by (*in situ*) small-angle and wide-angle X-ray scattering methods (SAXS and WAXS, respectively), especially using synchrotron-based X-rays with unmatched brilliance. Such experiments have proven indispensable for studying colloidal NCs.^45,46,56−61^

In this work, we monitored the entire lifespan of CsPbBr_3_ NCs in the TOPO/PbBr_2_ synthesis—from their nucleation to self-assembly into superlattices in the same reaction vessel—using synchrotron-based SAXS and *in situ* absorption spectroscopy. The synthesis was adjusted to ca. 30-fold higher concentrations (concentration of deficient species ca. (4–7) × 10^–3^ M) compared to those in ref (51), in line with common synthesis conditions for perovskite NCs^1^ but retaining sufficiently slow overall kinetics for a complete characterization. We followed the formation of the NCs, proceeding via an extended nucleation (up to 10% of the total reaction time) and size-focusing growth, for the whole length of the synthesis, thus enriching our understanding of the formation process. We identify the coordinating ligands (i.e., DOPA vs oleates) as the key parameter determining the stability of the Cs[PbBr_3_] solutes present as 3 nm large dispersed agglomerates, responsible for slow nucleation and growth. Moreover, even with oleate ligands we show that dilution can result in slow growth with the emergence of a series of magic-sized NCs. We also observe the spontaneous organization of the NCs in the reaction vessel, resulting in three-dimensional face-centered orthorhombic superstructures and give a full description of the self-assembly process.

## *In Situ* Time-Resolved SAXS: Cs-DOPA Case

**Figure 1:**
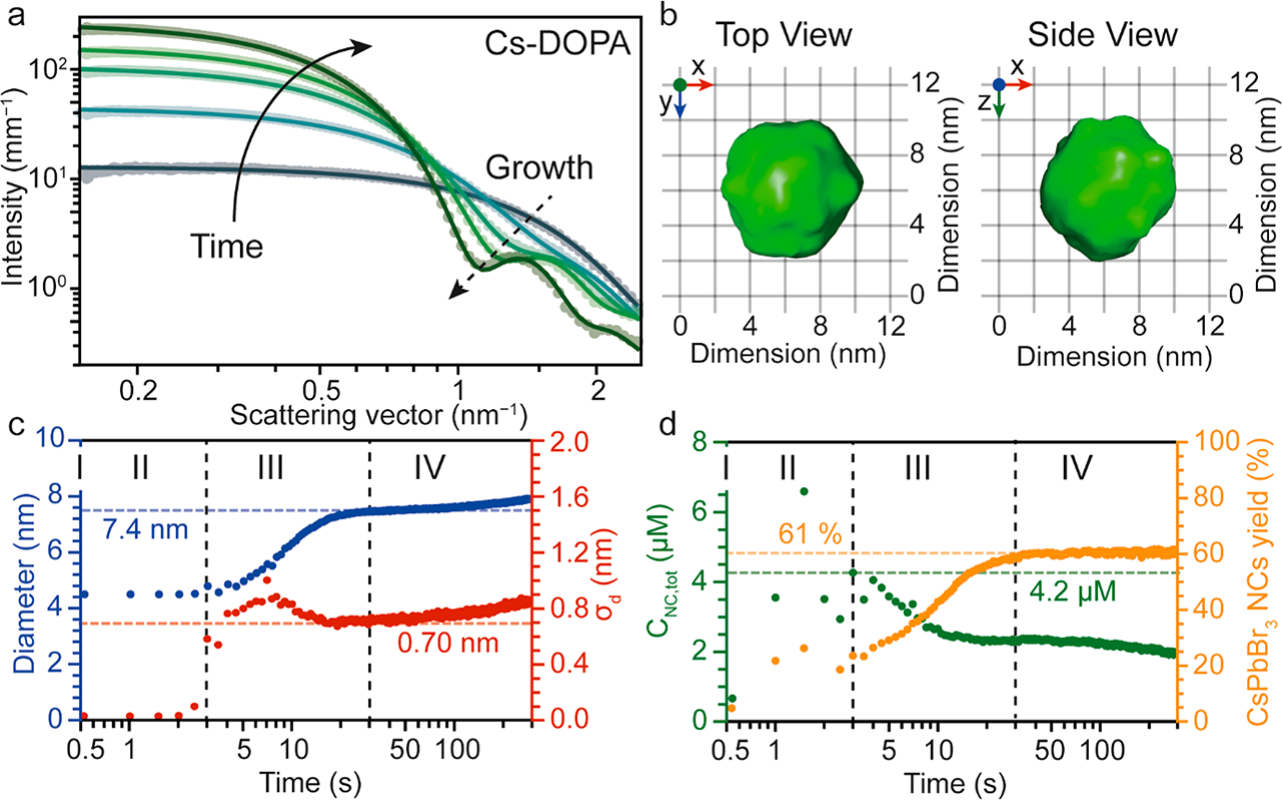
*In situ* time-resolved synchrotron-based SAXS results of the TOPO/PbBr_2_ synthesis. (a) Five representative SAXS patterns (data points) with their corresponding fits (solid lines) collected during the formation of CsPbBr_3_ NCs at 0.5, 5.5, 10.5, 15.5, and 300 s, respectively. The formation of NCs uniform in size manifests itself as local minima at higher values of scattering vector **q**. (b) Average particle model obtained by independently fitting the last scattering curve (*t* = 300 s) with a shape-retrieval, dummy-atom-model based algorithm.^62^ The particles are characterized by a quasi-spherical (rhombicuboctahedral) shape and a size of ∼8.1 nm. (c) Average size of the nanocrystals (blue) and standard deviation (red) as a function of the reaction time. The Roman numbers indicate the different phases of the synthesis. (d) NC concentration (green) and CsPbBr_3_ yield (orange; calculated on the yield of the deficient species, i.e. Cs) as a function of the reaction time. The two dotted vertical lines separate the different phases of the NC formation. The Roman numbers indicate the different phases of the synthesis.

The synthesis was adopted from our recent report,^51^ albeit for a 10–30 times higher overall concentration of all reagents (see the Supporting Information for further details). PbBr_2_ solubilized in a hexane solution of TOPO was reacted with Cs-DOPA. The synthesis was monitored *in situ* by recording small-angle and wide-angle X-ray scattering (SAXS and WAXS) every 0.5 s (beamline P21.2 at Petra III (DESY) in Hamburg; X-ray energy of 37.5 keV). Interestingly, already 0.5 s after injection (Figure 1a) 3.0 nm small particles are present in high concentration, which we identify as agglomerates of Cs[PbBr_3_] monomers. These species are characterized by the absorption band at 3.85 eV in ref (51), characteristic of zero-dimensional discrete PbBr_3_^–^ ions,^63,64^ and might be solubilized by TOPO. *In situ* WAXS shows the amorphous nature of these species (Figure S4). Over time, a population of larger particles (starting with size 4.4 nm), henceforth referred to as NCs, steadily emerges, seen as defined minima on top of the scattering pattern of the Cs[PbBr_3_] agglomerates (dashed arrow in Figure 1a and Figure S3), accompanied by a reduction in the concentration of the monomer agglomerates. Hence, the agglomerates consisting of Cs[PbBr_3_] monomers and possibly coordinated by TOPO molecules serve as precursors for the subsequent nuclei.

**Figure 2:**
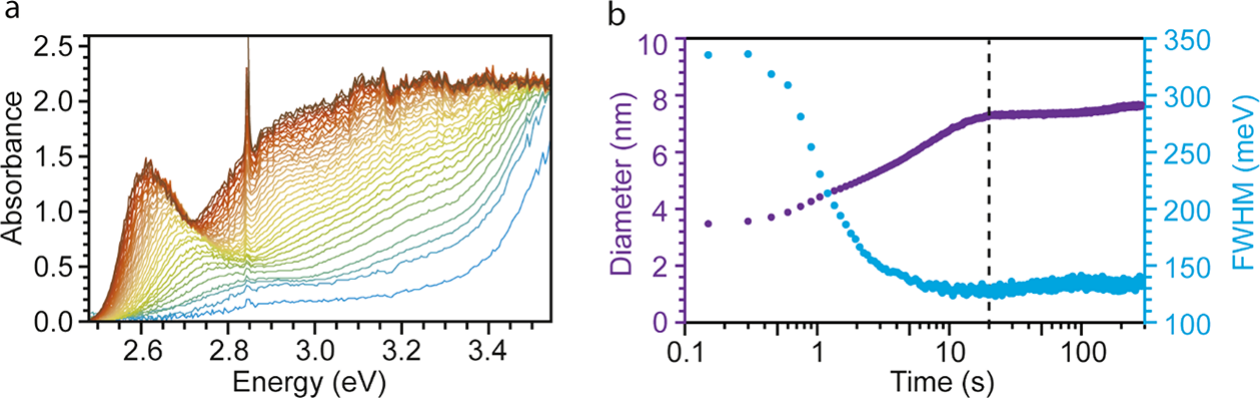
Monitoring nanocrystal growth by *in situ* optical absorbance spectroscopy. (a) Optical absorbance spectra collected during the synthesis of CsPbBr_3_ NCs with a time resolution of 150 ms. (b) Temporal evolution of the mean NC size (purple), determined from the excitonic peak energy using the calibration curve from ref (14) and full width at half-maximum (fwhm) (light blue) of the first excitonic peak. The as-obtained NC sizes agree very well with those determined from SAXS fits (blue curve in Figure 1c). The dotted line at 20 s marks the transition from the growth phase to Ostwald ripening.

From the final SAXS pattern, the average shape of the NCs was reconstructed (Figure 2b) by fitting the data with a shape-retrieval, dummy-atom-model based algorithm (see the Supporting Information); notably this fitting is performed without any prior assumption as to the shape of the scattering objects.^62^ The average particle is characterized by a quasi-spherical shape and an average size of 8.1 nm. This retrieved NC shape is in accordance with our previous report on TOPO/PbBr_2_ synthesis yielding rhombicuboctahedral-shaped NCs.^51^ The crystal structure of the NCs is confirmed to be orthorhombic CsPbBr_3_ (from WAXS, Figure S4; orthorhombic *Pbnm*, ICSD code 97851).

Four phases can be defined in the temporal evolution of the size, size distribution, concentration, and reaction yield (Figure 1c,d). In the first phase (*t* = 0.5 s, first recorded frame), the presence of 3.0 nm particles in high concentration was detected, showing that the conversion from PbBr_2_ precursors to the Cs[PbBr_3_] agglomerates of solutes, which occurs faster than the time resolution of the measurement (0.5 s), matches with the conversion of roughly 100 ms recorded via *in situ* absorption.^51^ In the second phase (*t* = 0.5–3 s), CsPbBr_3_ NCs of a discrete size of 4.4 nm form, whose population increases at the expense of 3.0 nm Cs[PbBr_3_] agglomerates (Figure S5a). When the concentration of the 4.4 nm CsPbBr_3_ NC peaks at 4.2 μM after 3 s (10% of the reaction time), the reaction enters the third phase (*t* = 3–30 s); here the 3.0 nm agglomerates are gradually depleted, while the 4.4 nm NCs grow at the expense of other (mostly smaller) NCs. In this phase, the NCs steadily decrease in number and polydispersity. The third phase concludes when the NC size plateaus at 7.4 nm, the polydispersity reaches a minimum of 0.70 nm (10% polydisperse; one CsPbBr_3_ monolayer), and the reservoir of Cs[PbBr_3_] agglomerates is completely depleted (Figure S5a); in this phase the reaction reaches its final yield (61%). Interestingly, the total mass of the Cs[PbBr_3_] solutes converts almost completely into the mass of the NCs (Figure S5b). In the last phase (*t* > 30 s), a moderate increase in the NC size and size distribution, concomitantly with a moderate decrease in NC number, is indicative of Ostwald ripening.

## *In Situ* Optical Absorbance: Cs-DOPA Case

The same synthesis was repeated while measuring, *in situ*, the optical absorbance (time resolution 150 ms; Figure 2), complementing the SAXS data. Interestingly, the first formed CsPbBr_3_ NCs have an average size of 3.8 nm, slightly smaller than that obtained by SAXS. However, the broad absorption feature, our reliance on calibration curves to extract the particle diameter (especially for small particle size), and the fact that the calibration was based on cubic particles can account for this discrepancy. The growth of the NCs is accompanied by a strong decrease in the full width at half-maximum (fwhm) from 340 to 135 meV (phase II of SAXS). During the subsequent period of 7–20 s, the NC size continuously increases until it plateaus at 7.6 nm, while the fwhm remains constant at 135 meV, evidencing the size-focusing behavior observed also by SAXS (phase III of SAXS). For longer times (*t* > 20 s), the size and the fwhm slowly increase, as expected for Ostwald ripening (phase IV of SAXS). At the end of the reaction, the NC number concentration was measured (via *ex situ* optical absorbance; see the Supporting Information for details) to be 1.1 μM, in agreement with SAXS measurements (Figure S6). Overall, the evolution of the NC size as recorded by *in situ* absorbance (purple curve in Figure 2b) is in strong agreement with the same data recorded by *in situ* SAXS (blue curve in Figure 1c and Figure S7).

When repeating the same experiment in a more diluted (×2.5) regime (Figure S8), to achieve a much broader spectral range, a strong absorption feature at 3.9 eV appears immediately after the injection (250 ms). This can be attributed to the conversion of the Cs-DOPA and PbBr_2_ precursors into Cs[PbBr_3_] agglomerates, as previously observed,^51,63,64^ confirming our observations by means of SAXS.

## Role of the Coordinating Ligands: Cs-OA

**Figure 3:**
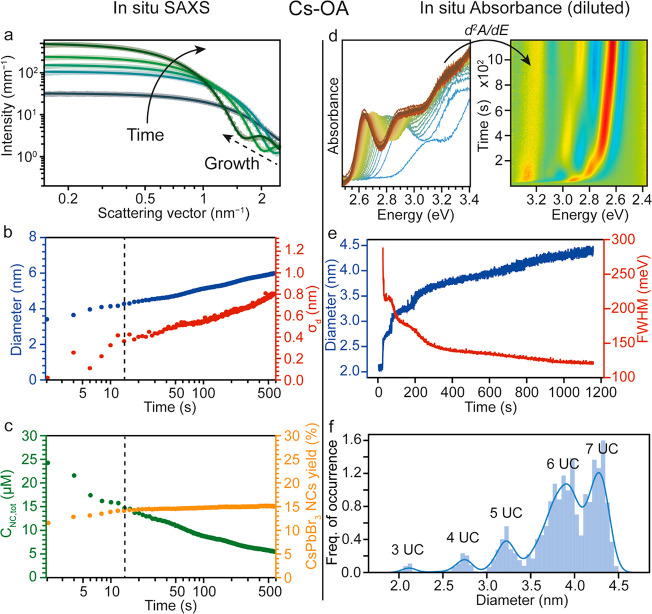
*In situ* time-resolved SAXS and optical absorbance measurements monitoring the evolution of the reaction using Cs-OA as the cationic reactant. (a) Five representative SAXS patterns (data points) collected during the formation of the nanocrystals, with their corresponding fits (solid lines). The patterns are collected at 2, 4, 12, 60, and 600 s, respectively. Over time the growth appears as a defined minimum shifting toward smaller values of the scattering vector **q**. (b) Average size (blue) and standard deviation (red) of the NC population as a function of the reaction time as extracted from the SAXS data. The dotted vertical line indicates the moment at which the reaction reaches its final yield. (c) Nanocrystal concentration (green) and CsPbBr_3_ yield (orange) as a function of the reaction time. The dotted vertical line indicates the moment at which the reaction reaches its final yield. (d) (left) Optical absorbance spectra collected *in situ* during the synthesis of CsPbBr_3_ NCs with Cs-OA (time resolution 100 ms). The peak associated with the first excitonic transition evolves over time, shifting toward lower energies, thus indicating NC growth. (right) Heat map representing the evolution over time of the second derivative of the absorption spectra, highlighting the discrete growth of the NCs over a series of very monodisperse (magic-sized) NCs. (e) Average size of the NCs (blue) as extracted from the position of the first excitonic peak and fwhm of the first excitonic peak (red) as a function of time. The abrupt shifts in both trends are symptomatic of the discrete growth of the NCs under these diluted conditions. (f) Histogram of the NC size as extracted from *in situ* absorption data over time using a calibration curve. The values above are the estimated diameters of the NCs in number of unit cells (UC), determined from the excitonic peak energy using the calibration curve from ref (14).

The choice of coordinating ligand has a pivotal role in determining, among other things, the reaction kinetics and formation mechanism of NCs. In our synthesis, DOPA, introduced in the synthesis as Cs-DOPA, acts as a stabilizer for the obtained NCs, as follows from NMR (Figure S9). For comparison, we ran the synthesis substituting Cs-DOPA with Cs-oleate (Cs-OA) and acquired SAXS data. In contrast to the synthesis with Cs-DOPA, where the minima evidencing the formation of NCs appeared at a specific value of the scattering vector (**q**), in this case the minima shift from larger to smaller values (dashed arrow in Figure 3a). The obtained NCs have a similar rounded shape and the same crystal structure (Figures S10 and S11) as in the Cs-DOPA case. Considering now the temporal evolution of the synthesis (Figure 3b,c), the first frame (2 s) shows the presence of CsPbBr_3_ NCs in high concentrations (24 μM; ca. 3.4 nm in diameter; Figure S11) and the absence of any free Cs[PbBr_3_] agglomerates, evidencing a fast nucleation event (<2 s); at the same time, the reaction yield is ∼76% of its final value already at this early stage. The difference between the two syntheses becomes more evident at longer times (*t* > 2 s): for Cs-OA, the scattered signal originates from a single population of growing scattering objects, unlike that from the Cs[PbBr_3_] agglomerates and NC mixtures in the Cs-DOPA case. Furthermore, for the Cs-OA case, the NC growth is accompanied by a decrease in the NC concentration and widening of the size distribution. Compared to Cs-DOPA, where size focusing is observed, the synthesis with Cs-OA produces a mere translation of the size distribution over time, without size focusing. At *t* = 14 s, the reaction reaches its final yield; this is notably only ∼25% of the final yield observed when Cs-DOPA is used. At this point the reaction enters the Ostwald ripening phase. The absence of Cs[PbBr_3_] agglomerates and size focusing and the presence of a fast nucleation event (<250 ms) are confirmed by *in situ* optical absorbance (Figures S12 and S13).

However, when performing the synthesis in much more diluted solutions (35–45 times), the *in situ* absorbance points to discrete steps in the evolution of the NC size (as extracted from the position of the first excitonic peak, see Figure 3d).These are accompanied by abrupt drops in the fwhm of the first excitonic peak (Figure 3e). Furthermore, when producing a histogram of the NC sizes as extracted from each frame (100 ms), specific “magic-size” crystals can be identified (Figure 3f), showing that the growth occurs plane by plane, the intermediate NCs being very monodisperse and stable. In this respect, the intermediate magic-sized NCs strikingly remain the same when the reaction conditions such as dilution are altered to adjust the final NC size (Figure S14). Hence, dilution of the reactants vs the amount of oleic acid is a new and promising route to obtain highly monodisperse OA-capped CsPbBr_3_ NCs of any size.

## Nucleation and Discrete Growth Mechanism

**Figure 4:**
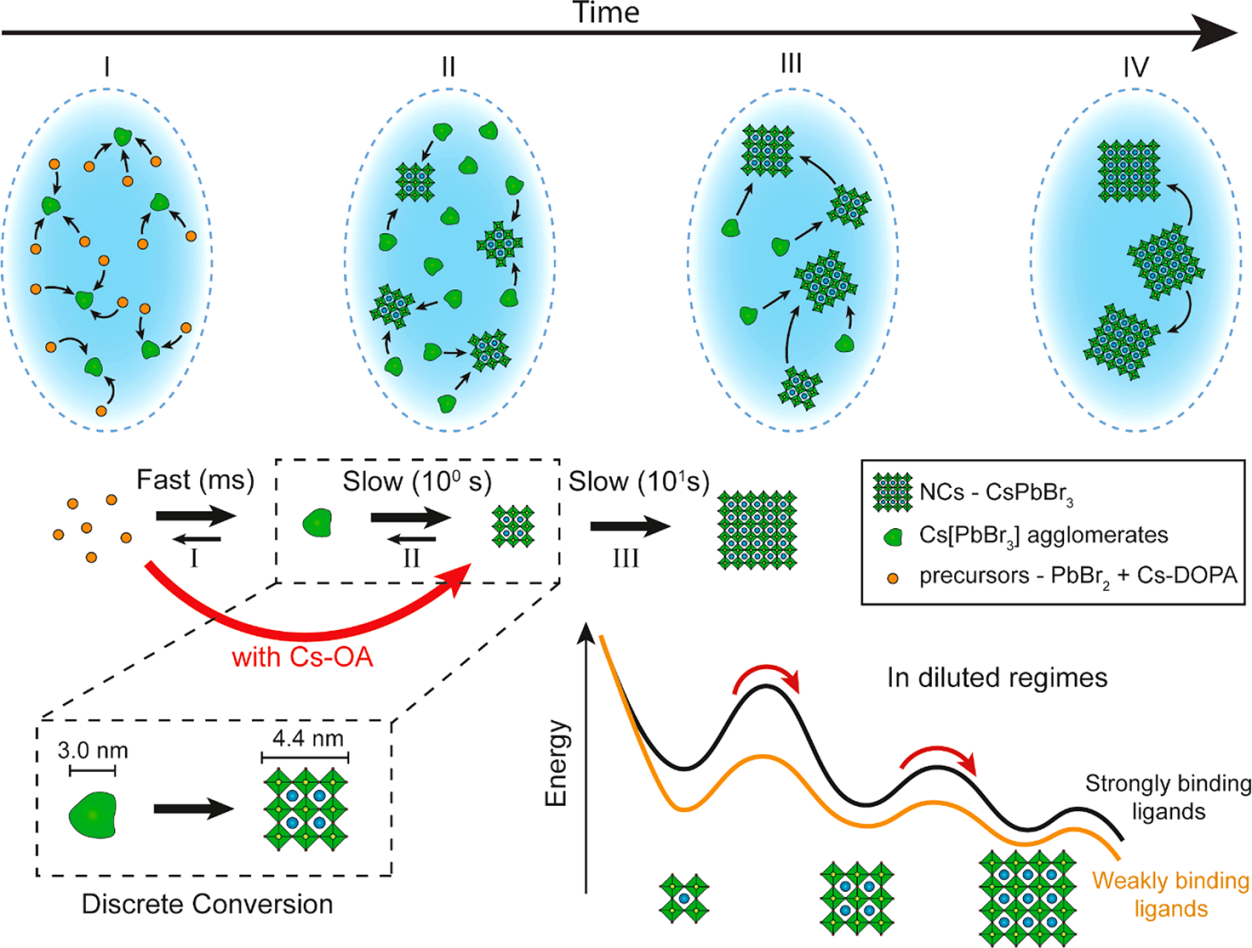
Schema showing the proposed formation mechanism for CsPbBr_3_ NCs involving the appearance of stable Cs[PbBr_3_] agglomerates and size-focusing growth when weakly binding ligands are used (i.e., DOPA). The NC formation proceeds through four phases: (I) very fast (approximately milliseconds) formation of 3.0 nm amorphous Cs[PbBr_3_] agglomerates from PbBr_2_ and Cs-DOPA precursors; (II) conversion of some 3.0 nm agglomerates into NCs with diameters as small as 4.4 nm (7 CsPbBr_3_ unit cells), where the NC concentration reaches a maximum; (III) size-focusing growth of the NCs at the expense of the Cs[PbBr_3_] agglomerates and via coalescence or redissolution of some of the NCs, with complete depletion of the 3.0 nm Cs[PbBr_3_] agglomerates; (IV) Ostwald ripening causing size defocusing and decrease in NC concentration. The use of Cs-OA as a precursor prevents the stabilization of the Cs[PbBr_3_] agglomerates and size focusing. The occurrence of discrete growth in diluted regimes is enabled by the energetically consistent kinetic barriers between NCs of different size, originating from the presence, in solution, of strongly binding ligands (i.e., OA).

The *in situ* optical absorbance and SAXS results presented above, together with our former study on the same system at much higher dilution levels,^51^ point to the following mechanism of NC formation (Figure 4). First, PbBr_2_ and Cs-DOPA rapidly convert into 3.0 nm amorphous agglomerates, consisting of Cs[PbBr_3_] monomers; this occurs on a sub-millisecond scale (phase I). Subsequently, some of these agglomerates convert into 4.4 nm CsPbBr_3_ NCs within seconds (phase II). The CsPbBr_3_ NCs continue growing at the expense of the amorphous agglomerates and the smaller NCs (phase III), followed by Ostwald ripening (phase IV). We hypothesize that the binding affinity of the DOPA ligand in this room-temperature synthesis is key for observing size focusing. Specifically, the weakly binding DOPA ligands allow the formation of Cs[PbBr_3_] agglomerates. As these agglomerates “store” the Cs[PbBr_3_] monomers and keep them out of the solution, they slow down nucleation and growth. As previously shown,^38,51^ DOPA is a weak ligand to all lead bromide species; hence, it inherently stabilizes the formation of Cs[PbBr_3_] monomers that agglomerate. Under these synthesis conditions, the conversion of these agglomerates of monomers into 4.4 nm NCs is much slower than that of the precursors into agglomerates. Hence, the temporal evolution of the agglomerates’ mass and the CsPbBr_3_ NCs’ mass have the same kinetics (Figure S5b). Therefore, the low monomer concentration in solution limits the rate of nucleation and growth, in a complementary way as previously observed in highly diluted systems.^51^ The slow release of Cs[PbBr_3_] monomers results in an extended nucleation event (up to 10% of the total reaction time; phase II), followed by slow and size-focusing growth (phase III). In stark contrast, when conventional strongly binding OA molecules are used,^44^ the stability of the Cs[PbBr_3_] is compromised (i.e., Pb(OA)_2_ is more stable than Pb(DOPA)_2_) and the formation of the NCs proceeds through a burst event (few milliseconds) and size defocusing.

In highly diluted regimes (concentration of deficient species ca. 9 × 10^–5^ M), the presence of strong binding ligands (OA) on the surface of the NCs gives origin to atomically discrete NC growth, as the energetically consistent kinetic barrier separating NCs of different size reflects the amount of energy required to remove a ligand and adsorb a new monomer.

## Spontaneous Assembly of Lead Halide Perovskite NCs in the Reaction Vessel

**Figure 5:**
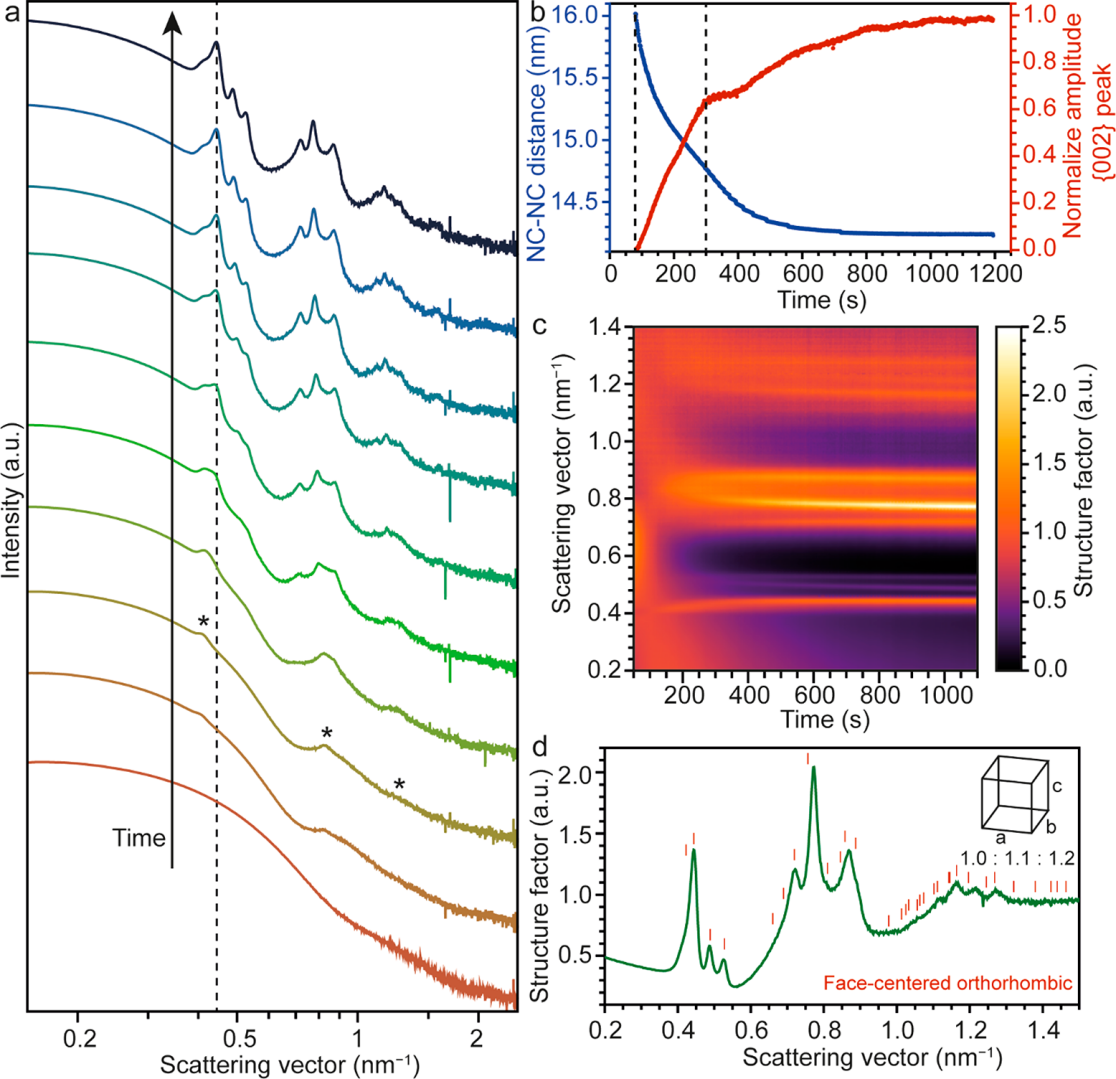
Self-assembly of lead halide perovskite NCs in solution. (a) SAXS patterns collected at different times (from the bottom to the top: 10, 90, 105, 200, 280, 420, 520, 680, 900, and 1200 s) during the spontaneous self-assembly of lead halide perovskite NCs in solution and shifted for clarity. The appearance of peaks at 0.39, 0.80, and 1.20 nm^–1^, indicating the alignment of NCs into one direction, after 90 s from the beginning of the reaction is highlighted by asterisks. Over time the peaks evolve into sharp structure factor peaks. The dotted line is a guide to appreciate the shift of the {002} structure peak toward higher values of **q** over time. (b) Evolution of the NC–NC distance (blue) and average amplitude of the {002} peak (red) over time. The two dotted lines highlight respectively the beginning of the crystallization (left line) at 90 s and the splitting of the three periodic peaks into multiple structure factor peaks (right line) after 300 s. (c) Heat map representing the evolution of the structure factor as a function of time. (d) Structure factor of the last recorded frame at 1100 s. The red ticks indicate the reference reflections of a face-centered orthorhombic crystal with aspect ratio 1.0:1.1:1.2.

Polydispersity of the NC size distribution and the choice of the ligand shell and the solvent (i.e., the strength of the interparticle forces) have a pivotal role in the interactions between NCs and NC self-assembly.^65^ When performing the synthesis at 100 °C with Cs-DOPA as a precursor, we observe, by means of *in situ* SAXS and in contrast to when Cs-OA is used (Figure S15), the appearance of sharp structure factor peaks, indicating the spontaneous formation of superstructures^11,13,66^ in the reaction vessel (Figure 5a). In the first 90 s of the reaction, the average NC diameter steadily grows to a value of 11.6 nm, comparable to the synthesis with Cs-OA (Figure S15). At this critical size, structure factor peaks arise at 0.39, 0.80, and 1.20 nm^–1^, which become sharper over time. The periodicity of the peaks suggests that the interparticle forces drive the NCs to assemble in a one-dimensional structure with a periodicity, or center-to-center distance, of 16.0 nm, similar to earlier observations with CdSe nanoplatelets.^67^ We suggest that the self-assembly into 1D structures is induced by the selective attachment of DOPA ligands to specific facets of the rhombocubioctahedral LHP NCs that are differently terminated. Over time, the NC–NC distance decreases (blue in Figure 5b) as the NCs are pulled together and their ligands start to interpenetrate; at the same time, the increase in sharpness of the first structure peak (red in Figure 5b) suggests that the average size of the one-dimensional superstructures is still increasing. 300 s into the reaction, the three periodic peaks evolve into multiple structure factor peaks whose amplitude and position change over time (Figure 5c). This suggests that the different one-dimensional superstructures combine to form a three-dimensional superstructure, as observed e.g. when semiconductor nanoplatelets are destabilized by the addition of an antisolvent.^67^ Notably, the three-dimensional crystallization happens when the NCs have an average NC–NC distance of 14.8 nm, hence with a surface-to-surface distance of 3.2 nm. At this surface-to-surface distance, van der Waals attractions can trigger the superstructure crystallization. This is consistent with previous observations of spontaneous crystallization triggered by interparticle interactions.^59,68−70^

By analyzing the last frame of the structure factor, we can determine that the crystal structure in which the LHP NCs self-assemble is face-centered orthorhombic (Figure 5d) with crystal lattice parameters *a*, *b*, and *c* of, respectively, 14.6, 16.0, and 17.8 nm, corresponding to an aspect ratio of 1.0:1.1:1.2. NCs of these sizes typically pack into face-centered-cubic or hexagonal-close-packed structures, which are the most favorable packing structures for spheres,^71,72^ as these structures are favored by the entropy-driven assembly and the interparticle interactions. However, since our particles are quasi-spherical (as determined from particle reconstruction, Figure 1b), their self-assembly results in a distorted face-centered cubic, hence face-centered orthorhombic, structure with an aspect ratio close to 1.0. The final size of the superstructures, as extracted from the fwhm of the SAXS data using the Scherrer equation , is ∼300 nm.

In summary, we investigated the formation of CsPbBr_3_ NCs in a TOPO/PbBr_2_ synthesis,^51^ recently reported to provide monodisperse NCs in a broad size range, by means of *in situ* SAXS and optical absorbance. The experimental evidence collected during the synthesis, which resulted in NCs with a highly monodisperse size distribution already in the crude product, allowed us to determine a general formation mechanism for this system, proceeding via extended nucleation events (3 s, ∼10% of growth time) and size-focusing growth. We identify dispersed agglomerates of Cs[PbBr_3_] monomers as the key intermediate species: they preclude the nucleation of NCs and slowly release Cs[PbBr_3_] monomers for further growth. Hence, the Cs[PbBr_3_] intermediate concentration is a key parameter in the reaction. This is also illustrated in the more commonly used synthesis with strongly binding OA ligands by simply diluting the reactants. Moreover, when the synthesis was performed at high temperatures (100 °C), spontaneous self-assembly of the NCs into three-dimensional superstructures was observed when the NCs reached a critical size of 11.6 nm. Via a quantitative analysis of the scattering data over time, we determined the self-assembly mechanism, which proceeds first via the formation of one-dimensional superstructures, which later combine to form a three-dimensional NC lattice. The final crystal structure was identified as face-centered orthorhombic with a crystal cell of aspect ratio ∼1.0.

## Supporting Information Available

Synthetic procedure for CsPbBr_3_ nanocrystals, *in situ* analysis by SAXS and optical absorption, data analysis of SAXS patterns, *in situ* WAXS data, and characterization methods (PDF)
