# Supplementary material for: Growth and Self-Assembly of CsPbBr3 Nanocrystals in the TOPO/PbBr2 Synthesis as Seen with X-ray Scattering
Source: Nano Lett. 2023 Jan 6;23(2):667–76. doi: 10.1021/acs.nanolett.2c04532 (PMC9881167; doi:10.1021/acs.nanolett.2c04532)
Supplement: Supplementary file 1 — nl2c04532_si_001.pdf [file nl2c04532_si_001.pdf]

Supplementary Information

Growth and Self-Assembly of $CsPbBr_3$ Nanocrystals in the TOPO/$PbBr_2$-Synthesis as seen with X-ray Scattering

Federico Montanarella[†,‡,*], Quinten A. Akkerman[†,‡], Dennis Bonatz[ǁ], Maaike M. van der Sluijs[§], Johanna C. van der Bok[§], P. Tim Prins[§], Marcel Aebli[†,‡], Alf Mews[ǁ], Daniel Vanmaekelbergh[§] and Maksym V. Kovalenko[†,‡,*]

† Laboratory of Inorganic Chemistry, Department of Chemistry and Applied Biosciences, ETH Zürich, Vladimir Prelog Weg 1, CH-8093, Zürich, Switzerland

‡ Laboratory for Thin Films and Photovoltaics, Empa − Swiss Federal Laboratories for Materials Science and Technology, Überlandstrasse 129, CH-8600 Dübendorf, Switzerland

§ Debye Institute for Nanomaterials Science, Utrecht University, 3584 CC Utrecht, The Netherlands

ǁ Institute of Physical Chemistry, University of Hamburg, 20146 Hamburg, Germany

$CsPbBr_3$ Nanocrystals Synthesis

Chemicals

Lead (II) bromide ($PbBr_2$, 98%), n-octane (99+% extra dry) and hexane (anhydrous) were purchased from Acros Organics. Cesium Carbonate (Cs_2CO_3, 99.9%), diisooctylphosphinic acid (DOPA, technical 90%) and oleic Acid (OA, 90%) were purchased from Sigma Aldrich. Trioctylphosphine oxide (TOPO, min 90%) was purchased from Strem Chemicals. All chemicals were used as received.

Cesium oleate (Cs-OA) 0.02 M

The preparation of cesium oleate was performed in a 40 mL vial, at room temperature and under ambient conditions. Cs_2CO_3 0.124 mmol (40 mg) was loaded in the vial along with OA 6.33 mmol (2 mL) and 10 mL of hexane or octane. The reagents were stirred with the aid of a magnetic stirrer until complete dissolution.

Cesium diisooctylphosphinate (Cs-DOPA) 0.02 M

The preparation of cesium diisooctylphosphinate was performed in a 40 mL vial, at room temperature and under ambient conditions. Cs_2CO_3 0.124 mmol (40 mg) was loaded in the vial along with DOPA 6.31 mmol (2 mL) and 10 mL of hexane or octane. The reagents were stirred with the aid of a magnetic stirrer until complete dissolution.

Lead (II) bromide precursor ($PbBr_2$-TOPO) 0.04 M

The preparation of cesium diisooctylphosphinate was performed in a 40 mL vial, at room temperature and under ambient conditions. $PbBr_2$ 0.4 mmol (147 mg) was loaded in the vial along with TOPO 2 mmol (773 mg) and 10 mL of hexane or octane. The reagents were stirred with the aid of a magnetic stirrer until complete dissolution.

Nanocrystals synthesis

In a typical synthesis, adapted from Ref.[1], the required amount (see Table below) of lead bromide solution was loaded in a 40 mL vial along with the required amount of hexane (e.g. for the synthesis of Figure 1 in the main text we used 3.5 mL of $PbBr_2$ solution along with 3.5 mL of hexane). The precursors were stirred with the aid of a magnetic stirrer. To trigger the formation of nanocrystals (NCs), we instantly injected a solution of Cs-OA or Cs-DOPA, in the required amounts (e.g. for the synthesis of Figure 1 in the main text we injected 1.75 mL of Cs-DOPA), by using a remotely-controlled liquid injector. The reaction was performed at room temperature in ambient atmosphere. For the high temperature reactions (i.e. 50 °C and 100 °C) we substituted hexane with octane. The reaction was probed via in situ X-ray scattering and usually terminated after a variable time (i.e. 5-30 min).

Table S1: Amounts of precursors used during the different experiments

Figure	Solvent type	Amount of solvent (mL)	Amount of $PbBr_2$ prec. (mL)	Amount of Cs precursor (mL)	Cs precursor type
Fig. 1-2, S3-7	Hexane	3.5	3.5	1.75	DOPA
Fig. 3 SAXS, S10-12	Hexane	4.5	4.5	2.25	OA
Fig. 3 Abs., S14 purple	Hexane	6.0	5.0	0.250	OA
Fig. 5, S15	Octane	3.5	3.5	1.75	DOPA
Fig. S8	Hexane	9.0	2.0	1.0	DOPA
Fig. S15	Octane	3.5	3.5	1.75	OA
Fig. S14 blue	Hexane	6.0	5.0	1.0	OA
Fig. S14 cyan	Hexane	3.0	2.5	0.5	OA

In situ X-ray scattering measurements

Data acquisition

The SAXS/WAXS experiment was performed at beamline P21.2 at Petra III (DESY) synchrotron in Hamburg. We used an energy of 37.5 keV (0.0331 nm) with VAREX XRD4343CT detector and a sample-to-detector distance of 1.5 m (for WAXS) and 15 m (SAXS). These distances allowed us to probe a **q** range between 16 nm^{-1} and 58 nm^{-1} for the WAXS and between 0.15 nm^{-1} and 2.2 nm^{-1} for the SAXS. A LaB_6 standard was used for calibration of the **q** range, of the scattering intensity and of the instrumental resolution, while absolute intensities were obtained by calibrating with glassy carbon and normalizing for the path length.[2] The acquisition time was typically 0.5 s or 2 s, depending on the dilution of the precursors.

Analysis of the SAXS data

For the analysis of the SAXS data, we modeled the scattering pattern of a dispersion of nanocrystals with an isotropic form factor and a Gaussian distribution. Based on former reports,[3] the shape of the nanocrystals is quasi-spherical (in contrast to conventional cesium LHP nanocrystals[4]), hence our choice to use a spherical form factor to describe our scattering objects. This assumption is also supported by the results of the fitting of the scattering curve with a shape-retrieval algorithm based on dummy atoms (Fig. 1b).
The scattering intensity is therefore expressed by the formula[5]:

$$I(q) = c_{NC,tot} N_A P_{sphere}(q, R)$$

Where, $c_{NC,tot}$ is the molar concentration of nanocrystals, N_A is the Avogadro number and $P_{sphere}(q)$ is the isotropic scattering form factor of a spherical scattering object, which is given by:

$$P_{sphere}(q,R) = 36\pi\Delta\rho^2 V_{sphere}^2 \frac{(\sin(qR) - qcos(qR))^2}{(qR)^6}$$

If we also implement the Gaussian distribution of radii R, the form factor will assume the form of:

$$\langle P_{sphere}(q,R)\rangle_R = 36\pi\Delta\rho^2 \frac{1}{\sqrt{2\pi\sigma_R}} \int_0^{\infty} e^{-\frac{1}{2}(\frac{R-R_0}{\sigma_R})^2} V_{sphere}^2 \frac{(\sin(qR) - qcos(qR))^2}{(qR)^6} dR$$

Where R_0 is the average radius of the distribution with standard deviation σ_R and $\Delta\rho$ is the scattering contrast, defined as the difference in the scattering length density of the nanocrystals and the solvent:

$$\Delta\rho = \rho_{CsPbBr_3} - \rho_{solvent}$$

The scattering length densities can be calculated with the following equation:

$$\rho = \frac{\delta\, 2\pi}{\lambda^2}$$

Where λ is the wavelength of the X-ray photons (0.331 Å) and δ is the real part of the refractive index of the material, which is expressed as $n = 1 + \delta - i\beta$. Since the imaginary part of the refractive index β is orders of magnitude smaller than the real part (since we do not work in the vicinity of an X-ray absorption edge), we neglect this part in the calculation of the scattering contrast. The values for δ can be calculated from the density of the material, and were obtained from the Centre for X-ray Optics (CXRO, https://henke.lbl.gov/optical_constants/) by extrapolating the values at 37.5 keV. The scattering length densities for the data analysis were obtained from two different online calculators (http://www.refcalc.appspot.com/sld and https://sld-calculator.appspot.com/save). The refractive index values used for the analysis were: $\delta_{CsPbBr_3} = 5.23\ 10^{-6}$ and $\delta_{solvent} = 7.75\ 10^{-7}$. The scattering length density used were: $\rho_{CsPbBr_3} = 3.1\ 10^{-5}$ Å^{-2} and $\rho_{solvent} = 4.5\ 10^{-6}$ Å^{-2} at 37.5 keV and 25 °C. This results in a scattering contrast of $2.6\ 10^{-5}$ Å^{-2}.

For the analysis of the SAXS patterns after injection of the precursors, the SAXS pattern acquired before the injection has been used as background. In Figure S2 we show one of the typical SAXS patterns acquired for our experiments before precursor injection. All the SAXS patterns acquired before precursor injection show the complete absence of any scattering object.

Estimation of error on experimental geometry

Due to the thickness of the probed sample (1 cm diameter vial), assumptions for a punctiform scattering object are debatable. Therefore, we estimated the effect of the spatial aberration on the signal acquired by the WAXS and SAXS detectors.

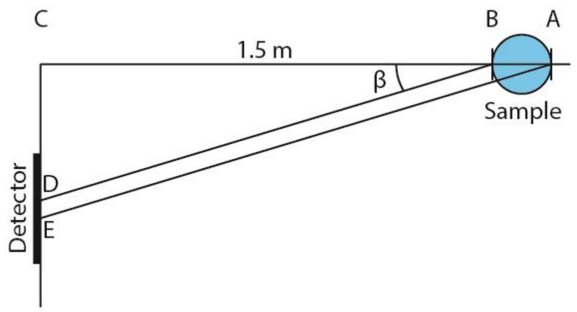

Figure S1: Geometry of the in situ SAXS/WAXS experimental setup.

Considering the geometry of Figure S1, the sample-to-detector distance is 150 cm ($\overline{BC}$) and 151 cm ($\overline{AC}$) respectively for the two ends of the sample (1 cm wide). The spatial aberration on the detector, $\overline{DE}$, can then be described as:

$$\overline{DE} = \overline{CE} - \overline{CD} = \overline{AC}\sin\beta - \overline{BC}\sin\beta = \overline{AB}\sin\beta$$

Considering the two extremes of β in our experiment, determined by the physical position of the WAXS detector, as 8° ($\overline{BD}$ and $\overline{AE}$) and 25° ($\overline{BE}$), the spatial aberration would be 0.14 cm and 0.42 cm respectively. Considering that each pixel is ~0.15 cm, the total error associated to spatial aberration at the two extremes of the detector will be between 0.1° and 0.3° respectively for 8° and 25°. Such small error implies that the assumption of a punctiform scattering object is still valid. The larger distance of the SAXS detector (15 m) leads to an even smaller error for this measurement.

Reaction yield calculation

After obtaining the molar concentration and size distribution of the $CsPbBr_3$ nanocrystals from the fitting of the scattering data, we extracted the average number of Cs^+ cations n_{Cs^+} in a nanocrystal from the ratio between the average nanocrystals volume and the volume of the $CsPbBr_3$ unit cell (0.602 nm):

$$n_{Cs^+} = \frac{\langle V_{nanocrystals}\rangle_R}{V_{CsPbBr_3}}$$

The reaction yield (RY) is then defined from the ratio between the total amount of Cs^+ cations incorporated in the nanocrystals and the amount of Cs^+ cation present in solution at the beginning of the reaction:

$$RY = \frac{n_{Cs^+}\ c_{NC,tot}}{n_{Cs^+,0}}$$

Here, $c_{NC,tot}$ is the total concentration of nanocrystals, as extracted from the SAXS data analysis, and $n_{Cs^+,0}$ is the initial molar concentration of the Cs-precursor.

Analysis of the self-assembled crystalline structure

In order to determine the crystal structure in which the nanocrystals self-assemble in solution, we scanned through different crystal structures in order of decreasing symmetry (i.e. cubic, hexagonal, tetragonal, orthorhombic). For each crystal structure we fitted the experimental peak positions to the allowed reflections with a weighted least square fitting procedure in a custom script, the variable being the lattice parameters. The

optimal result is obtained by fitting with a face-centered orthorhombic crystal structure with lattice parameters: 14.6, 16.0 and 17.8 nm. The position of each allowed reflection is calculated as:

$$q_{hkl} = \sqrt{\frac{h^2}{a^2} + \frac{k^2}{b^2} + \frac{l^2}{c^2}}$$

With h, k, l as Miller indices of the allowed reflections (i.e. reflections for which h, k, l are all odd or even) and a, b, c the lattice parameters.

The structure factor of the superstructures $S(q, R)$ was obtained by dividing each SAXS pattern $I(q, R)$ by the effective form factor $P(q, R)$, i.e. the scattering pattern of non-interacting nanocrystals before self-assembly. After indexing each experimental peak of the scattering pattern, we could extract the average NC-NC distance along the {002} direction by following the evolution of the position of the {002} peak ($PP_{\{002\}}$):

$$NC - NC\ distance = \frac{4\pi}{2PP_{\{002\}}}$$

Particle reconstruction

Particle reconstruction models were obtained by independently fitting the scattering curves with a commercially available algorithm based on dummy atoms (SasHel).[6]

NMR analysis

Solution ^{31}P NMR spectra were recorded on a Bruker 11.7 T spectrometer equipped with an AVANCE III console and a PABBO probe. A 30° excitation pulse (4.7 µs) and ^{1}H decoupling were used. ^{31}P chemical shifts were referenced externally relative to 85% H_3PO_4 in H_2O.

In situ optical absorbance measurements

In situ optical absorbance measurements were performed using a custom-made three-neck flask[7] equipped with an indentation, allowing to probe a small path length (1 mm) when performing the synthesis in high concentrations. The absorbance of the crude mixture was probed with an Ocean Optics deuterium-tungsten light source (DH-2000-BAL-TTL-24V) and an Ocean Optics OCEAN-HDX-XR spectrometer.

Ex situ optical absorbance measurements

Ex situ optical absorbance measurements were performed using a Jasco V670 spectrometer in transmission mode. The sample was injected in a 150 µm gap between two glued glass slides.

Additional Figures

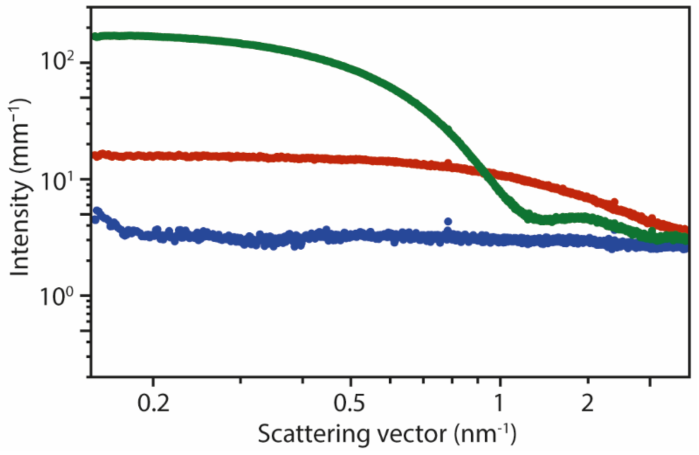

Figure S2. Not-background-corrected SAXS patterns. Not-background subtracted scattering patterns collected at −3.5 s (before precursor injection; blue dots), 1.5 s (red dots) and 21.5 s (green dots) during the synthesis of $CsPbBr_3$ NCs. The scattering pattern at −3.5 s shows the complete absence of any scattering object in solution prior to precursor injection. To better appreciate the features that arise after the injection, this SAXS pattern has been used as background and subtracted for all the subsequent SAXS patterns.

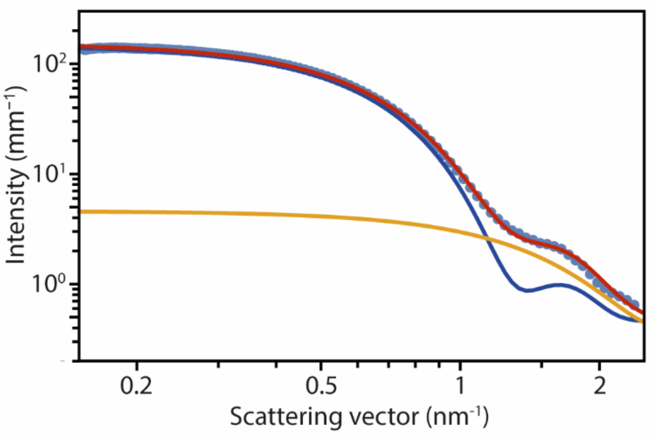

Figure S3. Contributions to the X-ray scattering signal. Scattering pattern (blue dots) collected after 15.5 s during the synthesis of $CsPbBr_3$ NCs and relative fit (red line). The fitting can be decomposed into two scattering contributions: one, present from the first frame after injection, and corresponding to Cs[$PbBr_3$] agglomerates of monomers with constant average size of 3.0 nm (yellow line), and the second one, whose size increases over time from the starting value of 4.4 nm, associated to $CsPbBr_3$ NCs (dark blue line).

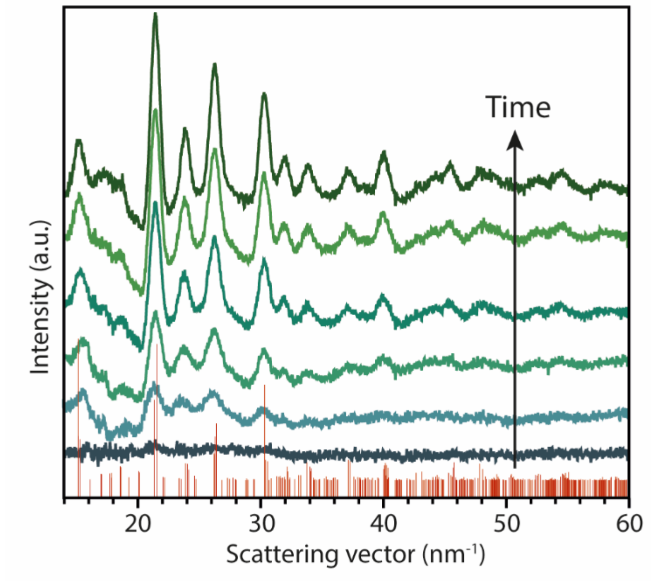

Figure S4. In situ wide angle X-ray scattering. Five representative WAXS patterns acquired during the formation of LHP NCs with Cs-DOPA as Cs precursor, shifted for clarity. The patterns are collected at 0.5 s, 2.0 s, 5.5 s, 10.5 s, 15.5 s and 300 s respectively. Over time the peaks become sharper, indicating an increase in the radius of the nanocrystals consistent with the SAXS data. The reference reflections for $CsPbBr_3$ are marked in red (orthorhombic Pbnm, ICSD code 978517).

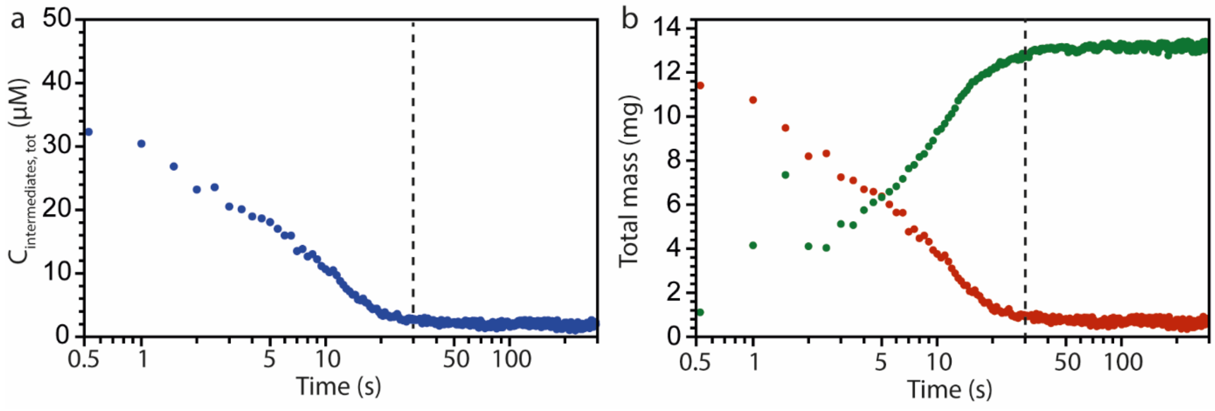

Figure S5. Evolution of the concentration of $Cs[PbBr_3]$ agglomerates over time. (a) Evolution of the concentration of the agglomerates of monomers (average size 3.0 nm) over time as extracted from the SAXS data. The dotted line indicates the moment at which the reaction reaches its final yield. (b) Evolution of the total mass for the $Cs[PbBr_3]$ agglomerates (red dots) and for the final NCs (green dots) over time. The dotted line indicates the moment at which the reaction reaches its final yield.

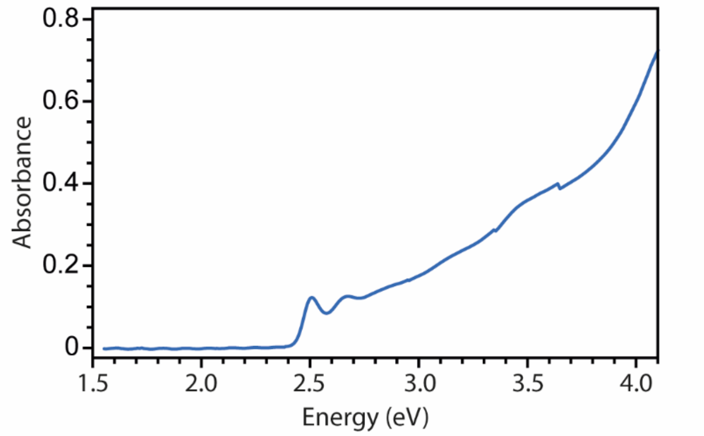

Figure S6. Optical absorption of the final crude synthesis product. In order to corroborate the value of final particle concentration as extracted from the SAXS data, we used a 150 μm glass slide to measure the absorption of the crude product at the end of the synthesis without having to dilute the product. The extracted concentration value (based on the absorption value at 3.7 eV)[8] for this sample was ~ 1.1 μM.

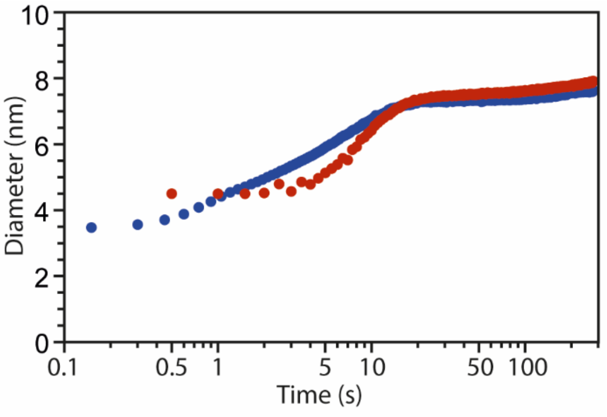

Figure S7. Evolution of NCs diameter. In order to better appreciate the agreement between the two characterization methods, we plot together the diameter of the NCs as function of time as recorded by means of SAXS (red; Fig. 1c) and optical absorbance (blue; Fig. 2b).

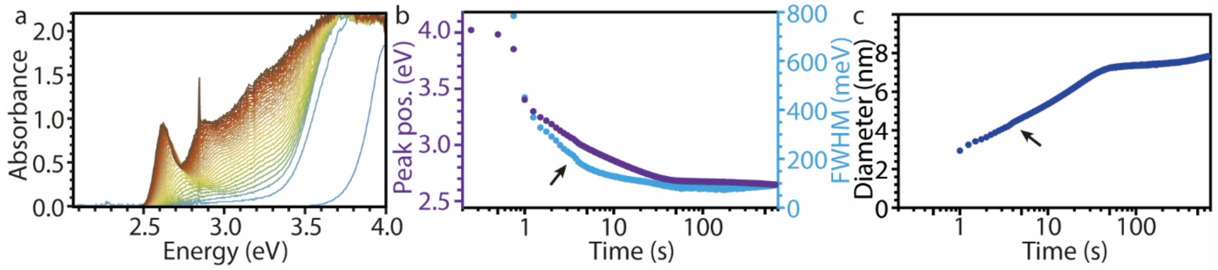

Figure S8. In situ optical absorption for a diluted synthesis. (a) Optical absorbance spectra collected in situ during the synthesis of $CsPbBr_3$ NCs with a time resolution of 250 ms (here plotted with resolution of 500 ms). Compared to the synthesis in Fig. 3 of the main text, this synthesis was 2.5x more diluted. The evolution of the first excitonic peak is more evident here thanks to the lower particle concentration. (b) Evolution of peak position (purple) and full width at half maximum (FWHM) (light blue) of the first excitonic peak over time, as extracted from the data of panel a. The abrupt shift of the peak position around 0.8 s indicates the conversion between Cs[$PbBr_3$] agglomerates (absorbing around 3.85 eV) and $CsPbBr_3$ nuclei (absorbing at 3.1 eV). The arrows indicate another (small) discontinuity in the data, corresponding to a peak position of 3.0 eV; this Energy corresponds to the absorption of NCs 4.4 nm in size, the smallest size observed for NCs by means of SAXS. From these observations we hypothesize that this discontinuity marks the end of the conversion of the agglomerates into 4.4-nm NCs. (c) Evolution of the average size of the nanocrystals in solution as extracted from the peak position in panel b. For the conversion we used a calibration curve from literature.[9] The arrow marks the position of the discontinuity marked in panel a, corresponding to a size of 4.4 nm.

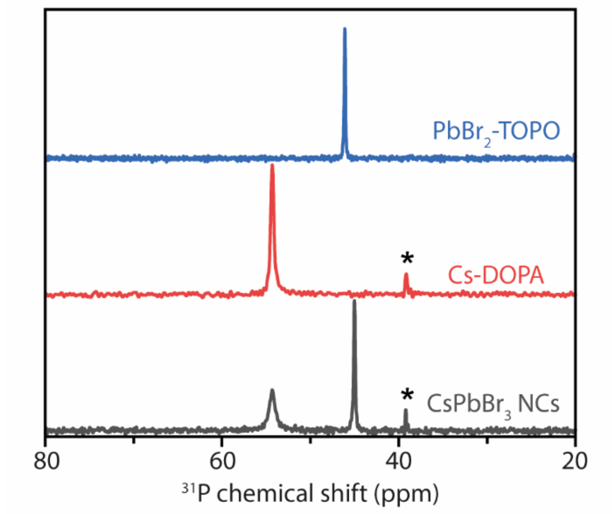

Figure S9. ^{31}P NMR of precursors and $CsPbBr_3$ NCs in solution. The ^{31}P NMR spectra of $PbBr_2$-TOPO (blue) and Cs-DOPA (red) show sharp features in agreement with the presence of unbound species in solution. For the $CsPbBr_3$ NCs in solution (grey) the signal associated to DOPA broadens, indicating the binding of this species to the formed NCs. Impurities are marked by asterisks.

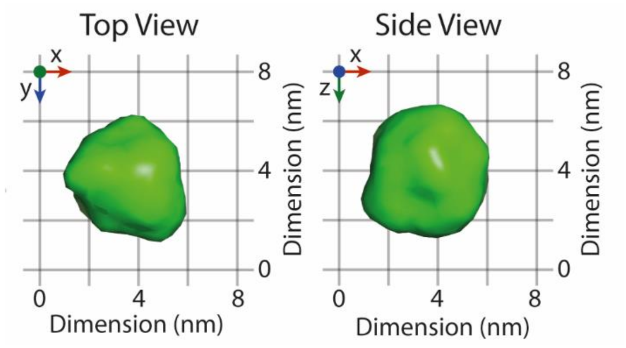

Figure S10. Particle reconstruction from SAXS data for synthesis with Cs-OA. Particle model obtained by independently fitting the last scattering curve (t = 600 s) with a shape-reconstruction, dummy-model-based algorithm.[6] The particles are characterized by a quasi-spherical shape and a size of ~5.2 nm.

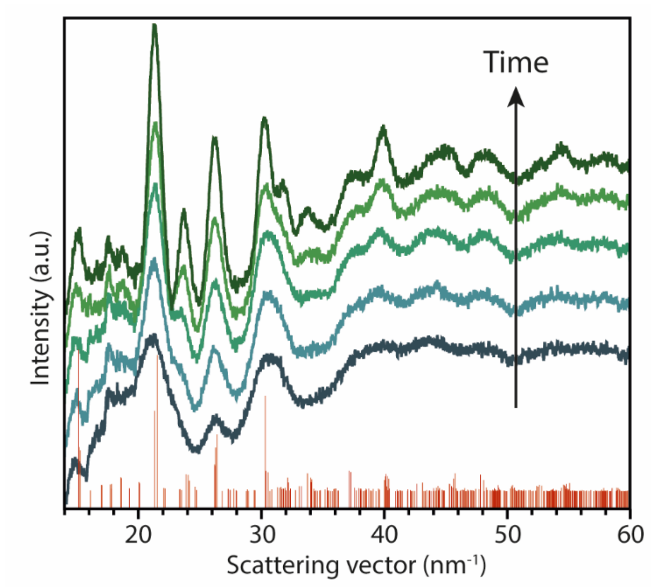

Figure S11. In situ wide angle X-ray scattering for synthesis with Cs-OA. Five representative WAXS patterns acquired during the formation of LHP nanocrystals using Cs-OA as Cs precursor, shifted for clarity. The color coding is the same as Fig. 4a in the main text: the patterns are collected at 2 s, 4 s, 12 s, 60 s and 600 s respectively. The reference reflections for $CsPbBr_3$ are marked in red (orthorhombic Pbnm, ICSD 97851).

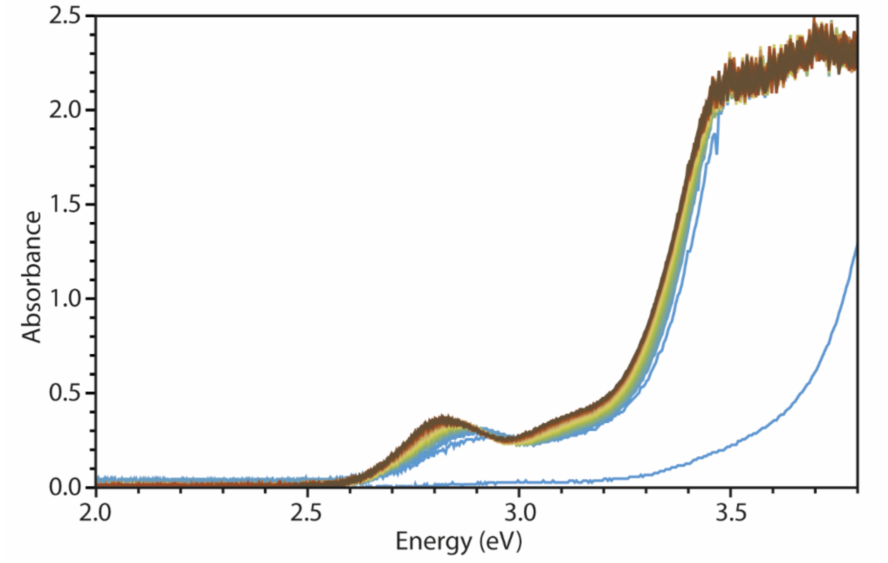

Figure S12. In situ optical absorption for synthesis with Cs-OA. Optical absorbance spectra collected in situ during the synthesis of $CsPbBr_3$ NCs when using Cs-OA as Cs precursor with a time resolution of 250 ms. The synthetic conditions are in all similar to the ones used for the data presented in Figure 2 of the main text, with the only difference that we used Cs-OA as Cs precursor instead of Cs-DOPA. We remark the striking difference to the absorption spectra of Figure 2: the appearance of excitonic features associated to NCs is here faster than the time resolution, pointing towards a fast (< 250 ms) nucleation event.

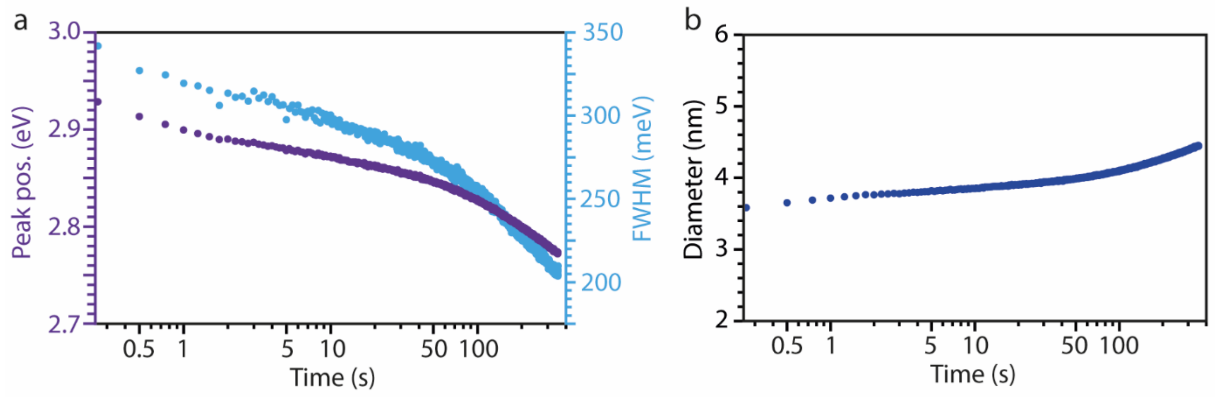

Figure S13. Synthetic parameters extracted from in situ optical absorption for synthesis with Cs-OA. (a) Evolution of peak position (purple) and full width at half maximum (FWHM) (light blue) of the first excitonic peak over time. (b) Evolution of the average diameter of the nanocrystals in solution as extracted from the peak position in panel a. For the conversion we used a calibration curve from literature.[9]

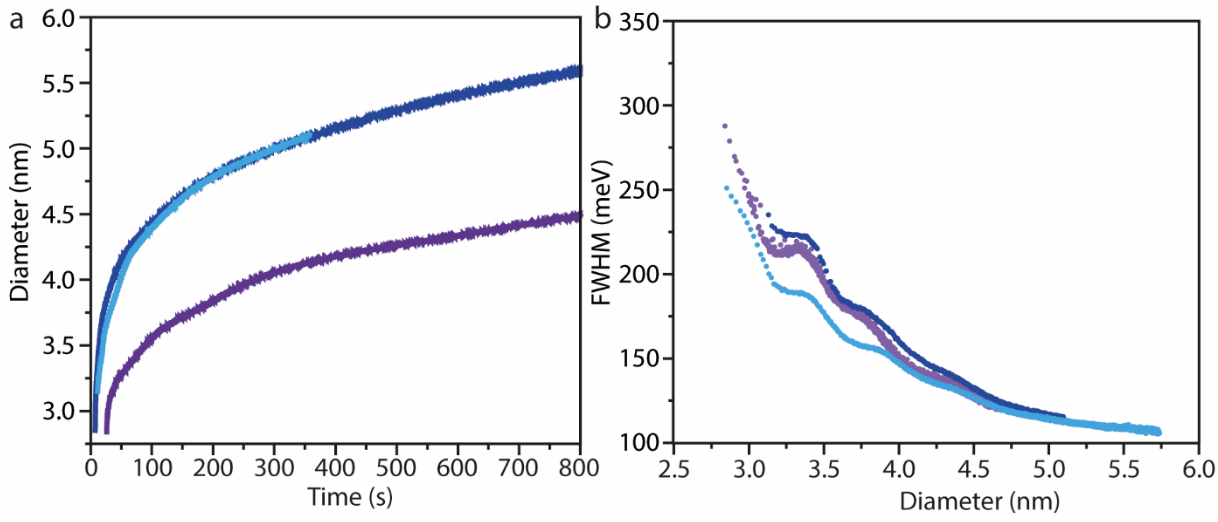

Figure S14. Discrete size growth for three syntheses with Cs-OA. (a) Evolution of NC diameter over time for three different syntheses with Cs-OA, as extracted from in situ absorbance measurements using a calibration curve.[9] (b) FWHM of the first excitonic peak as function of the NC diameter; both values were extracted from in situ absorbance measurements. Color coding correspond to the synthesis conditions of panel a. The oscillation of the FWHM, corresponding to discrete growth of the NCs, are independent of the synthesis conditions and of the final size.

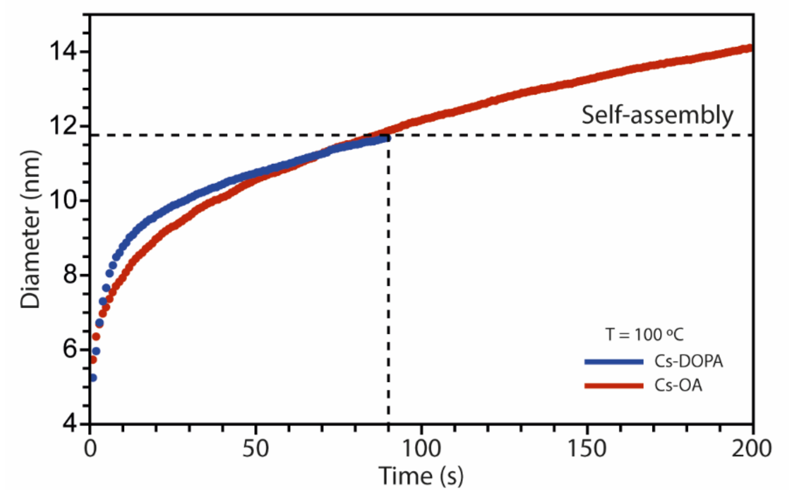

Figure S15. Evolution of the diameter during the formation of $CsPbBr_3$ NCs at 100 °C when using different Cs precursors. The synthesis was performed under the same reaction conditions in both cases, with the only difference being the Cs precursor: Cs-DOPA (blue) and Cs-OA (red). When the NCs are stabilized by Cs-DOPA they start to spontaneously self-assemble into bigger superstructures at the critical size of 11.6 nm. In contrast, when they are stabilized by Cs-OA, this behavior is not observed, despite growing to even bigger sizes.

References

(1) Akkerman, Q. A.; Nguyen, T. P. T.; Boehme, S. C.; Montanarella, F.; Dirin, D. N.; Wechsler, P.; Beiglböck, F.; Rainò, G.; Erni, R.; Katan, C.; Even, J.; Kovalenko, M. v. Controlling the Nucleation and Growth Kinetics of Lead Halide Perovskite Quantum Dots. *Science* **2022**, *377* (6613), 1406–1412. https://doi.org/10.1126/science.abq3616.

(2) Zhang, F.; Ilavsky, J.; Long, G. G.; Quintana, J. P. G.; Allen, A. J.; Jemian, P. R. Glassy Carbon as an Absolute Intensity Calibration Standard for Small-Angle Scattering. *Metallurgical and Materials Transactions A* **2010**, *41* (5), 1151–1158. https://doi.org/10.1007/s11661-009-9950-x.

(3) Almeida, G.; Ashton, O. J.; Goldoni, L.; Maggioni, D.; Petralanda, U.; Mishra, N.; Akkerman, Q. A.; Infante, I.; Snaith, H. J.; Manna, L. The Phosphine Oxide Route toward Lead Halide Perovskite Nanocrystals. *J Am Chem Soc* **2018**, *140* (44), 14878–14886. https://doi.org/10.1021/jacs.8b08978.

(4) Protesescu, L.; Yakunin, S.; Bodnarchuk, M. I.; Krieg, F.; Caputo, R.; Hendon, C. H.; Yang, R. X.; Walsh, A.; Kovalenko, M. v. Nanocrystals of Cesium Lead Halide Perovskites (CsPbX 3 , X = Cl, Br, and I): Novel Optoelectronic Materials Showing Bright Emission with Wide Color Gamut. *Nano Lett* **2015**, *15* (6), 3692–3696. https://doi.org/10.1021/nl5048779.

(5) Guinier, A.; Fournet, G. *Small-Angle Scattering of X-Rays*; John Wiley & Sons: New York, 1951.

(6) Burian, M.; Amenitsch, H. Dummy-Atom Modelling of Stacked and Helical Nanostructures from Solution Scattering Data. *IUCrJ* **2018**, *5* (4), 390–401. https://doi.org/10.1107/S2052252518005493.

(7) Prins, P. T.; Montanarella, F.; Dümbgen, K.; Justo, Y.; van der Bok, J. C.; Hinterding, S. O. M.; Geuchies, J. J.; Maes, J.; De Nolf, K.; Deelen, S.; Meijer, H.; Zinn, T.; Petukhov, A. V.; Rabouw, F. T.; De Mello Donega, C.; Vanmaekelbergh, D.; Hens, Z. Extended Nucleation and Superfocusing in Colloidal Semiconductor Nanocrystal Synthesis. *Nano Lett* **2021**, *21* (6), 2487–2496. https://doi.org/10.1021/acs.nanolett.0c04813.

(8) Maes, J.; Balcaen, L.; Drijvers, E.; Zhao, Q.; de Roo, J.; Vantomme, A.; Vanhaecke, F.; Geiregat, P.; Hens, Z. Light Absorption Coefficient of CsPbBr 3 Perovskite Nanocrystals. *J Phys Chem Lett* **2018**, *9* (11), 3093–3097. https://doi.org/10.1021/acs.jpclett.8b01065.

(9) Krieg, F.; Sercel, P. C.; Burian, M.; Andrusiv, H.; Bodnarchuk, M. I.; Stöferle, T.; Mahrt, R. F.; Naumenko, D.; Amenitsch, H.; Rainò, G.; Kovalenko, M. v. Monodisperse Long-Chain Sulfobetaine-Capped CsPbBr 3 Nanocrystals and Their Superfluorescent Assemblies. *ACS Cent Sci* **2021**, *7* (1), 135–144. https://doi.org/10.1021/acscentsci.0c01153.
